# Simple blood test may allow for early detection of oral cancer

**Published:** 2022-07

**JUNE 07, 2022 -** The current 60% 5-year survival rate of individuals with oral squamous cell carcinoma (OSCC) - a type of cancer of the mouth and throat - could be greatly improved if treatments were initiated as early as possible. In a study published in Natural Sciences, researchers used a technology called conductive polymer spray ionization mass spectrometry to screen the blood for metabolic signs of OSCC. The method could accurately distinguish between individuals with and without OSCC.

Also, 2 altered lipid markers that were discovered in the blood could be traced back to the cancer site for guiding surgical margin assessments.

The method - which requires only a single drop of blood - could also distinguish between patients with early versus later stages of OSCC.

“This study represents the fruits of research involving the United States and China, in which all the participants believed that the results would be a win for both countries and for the world as a whole, an idea that seems alas to be disappearing in these times of mutual suspicion and distrust of international collaborations,” said co–corresponding author Richard N, Zare, PhD, of Stanford University.

The author of an accompanying Research Highlight noted that the study “is a perfect showcase for how mass spectrometry-based metabolomics workflows can be simplified to make them usable in clinical applications.”

*Full Citation: “Big cohort metabolomic profiling of serum for oral squamous cell carcinoma screening and diagnosis” Xihu Yang, Xiaowei Song, Xudong Yang, Wei Han, Yong Fu, Shuai Wang, Xiaoxin Zhang, Guowen Sun, Yong Lu, Zhiyong Wang, Yanhong Ni, Richard N. Zare, Qingang Hu. Natural Sciences; Published Online: November 15, 2021 (DOI: 10.1002/ntls.20210071).*

*URL Upon Publication: https://onlinelibrary.wiley.com/doi/10.1002/ntls.20210071*

*Copyright © 2021 The Cochrane Collaboration. Published by John Wiley & Sons, Ltd., reproduced with permission.*

